# Flavonoid Intake and Plasma Sex Steroid Hormones, Prolactin, and Sex Hormone-Binding Globulin in Premenopausal Women

**DOI:** 10.3390/nu11112669

**Published:** 2019-11-05

**Authors:** You Wu, Susan E. Hankinson, Stephanie A. Smith-Warner, Molin Wang, A. Heather Eliassen

**Affiliations:** 1 Department of Nutrition, Harvard T.H. Chan School of Public Health, Boston, MA 02115, USA; swarner@hsph.harvard.edu; 2 Department of Biostatistics and Epidemiology, School of Public Health and Health Sciences, University of Massachusetts, Amherst, MA 01003, USA; shankinson@schoolph.umass.edu; 3 Department of Epidemiology, Harvard T.H. Chan School of Public Health, Boston, MA 02115, USA; stmow@channing.harvard.edu (M.W.); nhahe@channing.harvard.edu (A.H.E.); 4 Department of Biostatistics, Harvard T.H. Chan School of Public Health, Boston, MA 02115, USA; 5 Department of Medicine, Channing Division of Network Medicine, Brigham and Women’s Hospital and Harvard Medical School, Boston, MA 02115, USA

**Keywords:** flavonoid, sex hormones, biomarker, premenopausal women, circulating hormones

## Abstract

Background: Flavonoids potentially exert anti-cancer effects, as suggested by their chemical structures and supported by animal studies. In observational studies, however, the association between flavonoids and breast cancer, and potential underlying mechanisms, remain unclear. Objective: To examine the relationship between flavonoid intake and sex hormone levels using timed blood samples in follicular and luteal phases in the Nurses’ Health Study II among premenopausal women. Methods: Plasma concentrations of estrogens, androgens, progesterone, dehydroepiandrosterone (DHEA), DHEA sulfate (DHEAS), prolactin, and sex hormone-binding globulin (SHBG) were measured in samples collected between 1996 and 1999. Average flavonoid were calculated from semiquantitative food frequency questionnaires collected in 1995 and 1999. We used generalized linear models to calculate geometric mean hormone concentrations across categories of the intake of flavonoids and the subclasses. Results: Total flavonoid intake generally was not associated with the hormones of interest. The only significant association was with DHEAS (*p*-trend = 0.02), which was 11.1% (95% confidence interval (CI): −18.6%, −3.0%) lower comparing the highest vs. lowest quartile of flavonoid intake. In subclass analyses, the highest (vs. lowest) quartile of flavan-3-ol intake was associated with significantly lower DHEAS concentrations (−11.3% with 95% CI: −18.3%, −3.7%, *p*-trend = 0.01), and anthocyanin intake was associated with a significant inverse trend for DHEA (−18.0% with 95% CI: −27.9%, −6.7%, *p*-trend = 0.003). Conclusion: Flavonoid intake in this population had limited impact on most plasma sex hormones in premenopausal women. Anthocyanins and flavan-3-ols were associated with lower levels of DHEA and DHEAS.

## 1. Introduction

Flavonoids, a diverse group of polyphenols, consist of more than 5000 different compounds produced in plants [1,2]. They have been considered one of the active components in fruits and vegetables that have antioxidant, anti-inflammatory, and anti-proliferative properties [3].

The initial interest in studying flavonoids stemmed from the observation that Asian populations have higher intake of soy products, which are high in flavonoids, particularly isoflavones, and lower breast cancer incidence rates compared to Western populations. As circulating estrogens and androgens are positively associated with the risk for breast cancer in pre- and postmenopausal women [4,5,6,7], flavonoids are also of special interest in relation to breast cancer due to the structural resemblance to estrogens [8], yet its consequent risk and benefit are still controversial. In vitro studies have shown promising anti-cancer effects of flavonoids [9]. In observational studies, however, the association is less clear, as most significant associations have been reported in Asian populations, in case-control studies, or for postmenopausal breast cancer, but generally not in Western populations, prospective cohort studies, or for premenopausal breast cancer [10,11,12].

The discrepancies between in vitro studies and human studies suggest that flavonoids are involved in more complex biological processes in vivo, yet the direction in which flavonoids affect sex steroid hormone-dependent activities is far from conclusive. Soymilk that is rich in isoflavone reduces circulating ovarian steroids and adrenal androgens and increases menstrual cycle length in premenopausal women [13,14]; while other subclasses of flavonoid have been shown to have binding affinities to estrogen receptor-α (ERα), and possible stimulation of ER-dependent transcriptional response that promotes growth of estrogen-dependent cells was implicated [15,16,17]. The effect of total flavonoid intake on risk of breast cancer is a combined result of the relative strengths in either direction, as well as the overall effect of all subclasses; it is important to elucidate whether any subcalsses of flavonoid modulate circulating estrogen and other sex hormones. To date, no study has characterized the relationship between dietary intake of specific subclasses of flavonoids and blood sex hormone levels as they vary over the menstrual cycle. In this study, we investigated the relationship among premenopausal women using timed samples in both follicular and luteal phases in the Nurses’ Health Study II (NHSII).

## 2. Materials and Methods

### 2.1. Study Population

The NHSII is a prospective cohort study established in 1989 among 116,429 female registered nurses from 25 to 42 years of age. The enrolled women completed a questionnaire at study enrollment and are asked biennially to update with their medical and lifestyle factors and disease diagnoses. Between 1996 and 1999, blood samples were collected among a subgroup of participants in the NHSII. Details of the blood collection procedure are described in a prior publication [18]. Among the 29,611 participants who donated blood, 18,521 women who were not taking oral contraceptives, pregnant, or breastfed within 6 months providing timed samples collected in the follicular phase (3 to 5 days after the start of the menstrual cycle) and luteal phase (an estimated 7 to 9 days prior to the onset of the next menstrual cycle). Participants separated the plasma for the follicular sample and stored it in the freezer until the luteal sample was collected. Participants who provided untimed samples were included in analyses of dehydroepiandrosterone (DHEA), DHEA sulfate (DHEAS), testosterone, free testosterone, androstenedione, sex hormone–binding globulin (SHBG), and prolactin, as the concentrations of these hormones do not fluctuate much during menstrual cycles [19]. All blood samples were shipped overnight with an ice pack, processed by our laboratory into plasma, red blood cells, and white blood cells, and then stored in continuously monitored liquid nitrogen freezers (≤−130°). The original vials were thawed once to subaliquot a sample to send to each respective laboratory, and a second thaw occurred at the time of assay. The collection, processing, and storage methods of blood samples in NHSII have been shown to not materially alter levels of sex hormones. Furthermore, the between-person variation for most of the sex hormones far outweighed their within-person variation even with up to 2 days of delay in processing after phlebotomy [20,21].

Participants included in this analysis were part of a hormone reproducibility study [19], or were the controls in nested case-control studies of breast cancer [22], ovarian cancer [23], endometriosis [24], and rheumatoid arthritis [25]. A total of 2000 premenopausal women had measured concentrations of plasma sex hormones (estradiol, estrone, estrone sulfate, progesterone, DHEA, DHEAS, testosterone, androstenedione), prolactin, and SHBG concentrations. Among these participants, 1989 had flavonoid consumption data available in 1995, 1999, or both. The study protocol was approved by the Institutional Review Boards of the Brigham and Women’s Hospital and the Harvard T.H. Chan School of Public Health.

### 2.2. Laboratory Assays

The laboratory assay methods used to obtain the concentrations of each hormone were documented in prior publications [5,26]. Estrone, estradiol, and estrone sulfate concentration were assayed in luteal and follicular samples; testosterone, androstenedione, and prolactin concentrations were assayed in luteal and/or follicular, as well as untimed samples; DHEA, DHEAS, and SHBG were assayed in luteal and untimed samples; and progesterone was assayed in luteal samples only. The assays were performed in different batches at different laboratories for several different sets of nested case-control studies. Three batches of estrogens, five batches of testosterone, two batches of androstenedione, and one batch of progesterone were assayed at Quest Diagnostics (San Juan Capistrano, CA, USA). At this location, estrogens and testosterone were measured by organic extraction, celite column chromatography, and then radioimmunoassay (RIA); estrone sulfate was measured by extraction of estrone, enzyme hydrolysis, column chromatography, and then RIA of estrone; progesterone were measured by organic extraction and RIA. Four batches of estrogens and testosterone were assayed at Mayo Medical Laboratories (Rochester, MN, USA) using liquid chromatography–tandem mass spectrometry (LC-MS/MS). Two batches of DHEA and androstenedione and four batches of DHEAS, SHBG, and progesterone were assayed at the Royal Marsden Hospital (London, United Kingdom). At this location, androstenedione was measured by RIA; and DHEAS, SHBG, and progesterone were measured by chemiluminescent enzyme immunoassay. One batch of progesterone, three batches of SHBG, and prolactin were assayed at Massachusetts General Hospital (Boston, MA, USA), where progesterone was measured by RIA, SHBG was measured by chemiluminescent enzyme immunoassay, and prolactin was measured by microparticle enzyme immunoassay (AxSYM Immunoassay System). One batch of SHBG and progesterone was assayed at Boston Children’s Hospital (Boston, MA, USA). Batch correction was performed as descibed in the statistical method section.

Free and percent free estradiol were calculated by the law of mass action according to the method described by Sodergard et al. [27]. The detection limits of the assays were as follows: 2 pg/mL for estradiol, 10 pg/mL for estrone, 40 pg/mL for estrone sulfate (in each laboratory), 3 ng/dL for androstenedione, 1 ng/dL for testosterone, 3 ng/dL for DHEA, 5 μg/dL for DHEAS, and 3 ng/dL for progesterone. When plasma hormone values were reported as less than the detection limit, we set the value to half this limit. All plasma hormone concentrations under the detection limits were set to half of the minimum detection value.

The reproducibility of plasma estrogens (intraclass correlation coefficients, ICC: 0.38–0.69), androgens (ICC: 0.56–0.94), progesterone (ICC: 0.29), prolactin (ICC: 0.64), and sex hormone binding globulin (ICC: 0.86) has been documented [19].

### 2.3. Exposure and Covariates Measurement

Flavonoid intake was calculated from semiquantitative food frequency questionnaires (FFQ) administered closest to the blood collection to represent the average intake around the time between 1995 and 1999. The participants were asked to estimate their usual intake of a list of ~131 food items, from which the intake of flavonoids were calculated using a database maintained by Harvard dietitians (available for request at: https://regepi.bwh.harvard.edu/health/nutrition.html) that is largely based on the United States Department of Agriculture (USDA) food content database [28]. In the study that validated the FFQ against dietary records, the Spearman rank correlation coefficient is 0.55 for energy-adjusted total flavonoid intake, and from 0.41 to 0.58 for flavonoid subclasses [29]. For this study, we used average energy-adjusted intake of the nutrients from 1995 and 1999, or a single measure in 1995 or 1999 if there was only one available (*n* = 192). We evaluated total flavonoids in our primary analyses. As secondary analyses, we examined 6 flavonoid subclasses-flavonols, flavanones, flavones, flavan-3-ols, anthocyanins, and isoflavones.

From the questionnaire completed at blood collection or the questionnaire immediately prior to blood draw, we obtained data on potential confounders for the association between flavonoid intake and plasma sex hormone concentrations: age at blood collection, smoking status, body mass index (BMI), height, age at menarche, usual menstrual cycle length, menstrual cycle pattern, parity, family history of breast cancer, history of biopsy-confirmed benign breast disease, physical activity, date and time of day of blood collection, fasting status, luteal day of blood collection, average alcohol consumption, weight change from age 18, and total energy intake.

### 2.4. Statistical Analyses

Estradiol, free estradiol, estrone, and estrone sulfate were evaluated in follicular and luteal phases; progesterone was only evaluated in luteal phase; DHEA, DHEAS, testosterone, free testosterone, androstenedione, and prolactin were measured using the average of follicular and luteal phases or from a single luteal or untimed sample. We identified statistical outliers in the hormone data based on the generalized extreme studentized deviate (ESD) many-outlier detection approach [30]; the outliers were excluded from analyses. For each of the hormones, no more than 8 outliers were excluded. To address differences that might arise from assays being performed at multiple locations in multiple batches, all hormones were recalibrated to have a comparable distribution between all batches using the method of Rosner and colleagues [31,32]. In brief, we made statistical adjustment where the concentrations were combined to represent an average batch, then regressed on age, BMI (the strongest predictor of hormone levels), and indicator variables for each batch. Within each batch, the hormone levels were recalibrated according to the difference between the coefficients for that batch and the average of the batch coefficients. The adjusted concentrations thus accounted for between-batch variability independent of varying distributions of age and BMI across batches.

In our primary analyses, we calculated the geometric mean of each hormone concentration by quartile of total flavonoid intake using generalized linear models, with marginal covariate distribution in the study population. A contrast comparing the highest to the lowest quartile of flavonoid intake was also estimated. Confounders included in the models were based on subject matter knowledge and prior literature—age at blood collection in years (continuous), BMI at blood collection in kg/m^2^ (<22.5, 22.5 to <25, 25 to <27.5, 27.5 to <30, ≥30), total energy intake in kcal/day (continuous), smoking (never smoker, past smoker with ≥5 years since quitting, past smoker with <5 years since quitting, current smoker of <15 cigarettes per day, current smoker of 15 cigarettes per day), alcohol use (non-drinker, 10 g/day, 10.1–20 g/day, >20 g/day), physical activity (<3, 3–<9, 9–<18, 18–<27, ≥27 metabolic equivalent of task (MET)-hours/week), duration of past oral contraceptive use (never, <4 years, ≥4 years), age at menarche (<12, 12–13, >13 years), age at first birth/parity (nulliparous, 1–2 children and age at first birth <25 years, 1–2 children and age at first birth ≥25 years, >3 children and age at first birth <25 years, >3 children and age at first birth ≥25 years). Covariates related only to the outcomes—date of blood collection (month/year, 1/97, 2/97–1/98, 2/98–1/99, after 2/99), time of day of blood collection (1–8 a.m., 9 a.m. to noon, 1–4 p.m., 5 p.m. to midnight), and fasting status (<10, ≥10 h)—were also included in the models to reduce extraneous variation. Models that included luteal or average of timed samples also were adjusted for the difference between luteal blood draw date and date of next menstrual period (3–7, 8–12, 13–17, 18–21 days, unknown/untimed). We also evaluated the linear trend of flavonoid intake using median of each quartile modeled continuously and calculated a Wald test statistic. In secondary analyses, we repeated the main analyses for each subclass of flavonoids-flavonols, flavanones, flavones, flavan-3-ols, anthocyanins, and isoflavones. We also conducted a priori sensitivity analyses by excluding women who had luteal progesterone level <400 ng/dL (*n* = 230, 11.6%) to restrict the analysis to women who donated blood samples during an ovulatory cycle.

## 3. Results

The mean age at blood draw of the 1989 participants was 42.7 years, and the mean BMI was 26.1 kg/m^2^. Flavonoid intake did not vary substantially by height, BMI at age 18 or at blood draw, or reproductive factors. Those who had higher intake of total flavonoids tended to be more physically active, and less likely to be current smokers (Table 1).

Table 2 shows the median concentration and 10th–90th percentile of the plasma sex hormones, prolactin, and SHBG. The Spearman rank correlation coefficients between the concentrations of the hormones, prolactin, and SHBG ranged from 0.0007 for follicular estradiol and androstenedione, to 0.88 for follicular estradiol and follicular-free estradiol.

In our study, the major sources of food for flavonoids were tea and apples, making up over 50% of the total flavonoid intake. The top contributing food items for the six flavonoid subclasses were flavonol (tea and onions), flavone (orange and orange juice), flavanone (orange and orange juice), flavan-3-ol (tea and apples), anthocyanin (blueberries and strawberries), isoflavone (tofu and soymilk) (Table S3). Flavna-3-ol had foods with the highest intake among the six subclasses. The correlations between the subclasses of flavonoid are shown in Supplemental Table S1. Flavonol and flavan-3-ol intakes were highly correlated, and since flavan-3-ol had the highest intake level, both were also highy correlated with total flavonoid intake (Spearman correlation coefficients 0.75–0.97, *p* < 0.0001). The correlations between other pairs of flavonoids subclasses were relatively weak (Spearman correlation coefficients <0.25) (Supplementary Materials Table S1).

In this study, total flavonoid intake was not significantly associated with any of the sex hormone concentrations except for DHEAS (*p* = 0.02); plasma DHEAS was 11.1% lower comparing the highest vs the lowest quartile of flavonoid intake (95% confidence interval (CI): −18.6%, −3.0%) (Table 3). The percent difference comparing the highest versus the lowest quartile for the remaining sex hormones ranged from −7.4% to 5.1%.

In secondary analyses, we examined the six subclasses of flavonoids. Similar to total flavonoids, only DHEA and DHEAS showed significant differences between the extreme quartiles of the intake of the subclasses of flavonoid (Table 4). Interestingly, DHEAS was significantly lower (−11.3% with 95% CI: −18.3, −3.7%, *p*-trend = 0.01) comparing the extreme quartiles of flavan-3-ol intake, however in contrast, DHEAS tended to increase significantly across quartiles of flavanone intake, although the difference between the highest and the lowest quartiles were only marginally significant (9.0% with 95% CI: −0.3%, 19.3%, *p*-trend = 0.04). Across the quartiles of flavan-3-ol intake, luteal progesterone concentration showed a significant increasing trend (*p* = 0.03), although the contrast between the highest and the lowest quartile was not statistically significant (−7.6%, 95% CI: −14.78%, 0.20%). Anthocyanin intake was associated with significant inverse trends for DHEA (−18.0% with 95% CI: −27.9%, −6.7%, *p*-trend = 0.003) and follicular estrone sulfate, although the contrast between the extreme quartiles was not statistically significant for follicular estrone sulfate (−16.9% with 95% CI: −32.5%, 2.4%, *p*-trend = 0.02). Hormones with concentrations significantly higher comparing the highest and the lowest quartile of isoflavone were follicular estrone sulfate (25.7% with 95% CI: 3.6%, 52.6%) and DHEAS (12.7% with 95% CI: 3.5%, 22.8%), although neither had significant linear trend across quartiles of isoflavone intake.

In sensitivity analyses excluding anovulatory women (11.3%, *n* = 203), the results did not change materially. Total flavonoid intake remained significantly associated with DHEAS (*p*-trend = 0.04). Among the six subclasses of flavonoids, associations that remained significant were between and flavan-3-ols and DHEAS (*p*-trend = 0.03), and between anthocyanins and DHEA (*p*-trend = 0.01).

## 4. Discussion

In this study of 1989 premenopausal women, we did not observe strong associations between intake of total flavonoids and/or its subclasses and most of the sex hormones examined. The only significant trends were that women with higher flavonoid intakes, especially that of flavan-3-ol and anthocyanins, had lower plasma DHEA and DHEAS concentrations; this has been associated with decreased risk of premenopausal breast cancer in the same cohort [33], as well as in a large pooled analysis [6].

Flavonoids in general have been considered as phytochemicals with the potential for antioxidant and anti-inflammatory activity [34,35], although the antioxidant capacity is limited after extensive metabolism that is sufficient to reduce plasma indices of oxidant status in vivo. In vitro studies suggest that they also have the promise as anti-tumor agents [3]-a number of flavonoids can induce apoptosis in cancer (including breast cancer) cell lines, arreste proliferation at the G2/M cell cycle checkpoint [36,37], and bound strongly with Estrogen Receptor β, (ER-β), which opposes the proliferative effects of ERα activation [38]. However, due to their estrogenic feature, some flavonoids also have binding affinity to ER-α, indicating possible promotion of proliferation [16]. The health benefit of even the most promising subclass-isoflavone-was challenged, given that soy isoflavone extracts may promote estrogen receptor positive breast cancer cell growth [39]. In addition, the majority of the evidence from lab-based experimental studies did not take into consideration the absorption and metabolic interactions within the human body. Epidemiologic studies that examined the relationship between flavonoids (dietary intake and circulating concentrations, as well as urinary levels) and breast cancer risk among humans have shown inconsistent results so far [11]. For example, short-term supplementation of soymilk rich in isoflavones decreased circulating estrogens among premenpausal women, and isoflavones were associated with lower premenopausal breast cancer risk in Asian and German populations [40]. However, studies also suggest that soy (the main source of isoflavone) supplementation may increase the expression of several tumor-promoting genes [41], and the breast cancer risk reduction by isoflavone was not seen in most Western countries.

A meta-analysis published in 2013 synthesized multiple prospective cohort and case-control studies on flavonoid intake and breast cancer risk. While total flavonoid intake, especially that of flavonols and flavones, was inversely associated with postmenopausal breast cancer, the results for premenopausal women were non-significant [42]. Among all proposed effects of flavonoid, most (e.g., antioxidation, anti-inflammation, inhibition of carcinogen-activating enzymes) are independent of menopausal status. What is most plausible to explain the different effects of flavonoid on pre and postmenopausal breast cancer is the complicated interaction between flavonoid and sex hormones. One may be concerned that flavonoids might alter sex hormones in premenopausal women in the undesired direction thus counteract the overall benefit. Our results are reassuring that flavonoids are not associated with higher concentrations of circulating sex hormones. In fact, DHEA and DHEAS demonstrated inverse linear trends with higher intake of anthocyanins and flavan-3-ols. In vitro studies have shown that DHEA increases proliferation in the human breast cancer cell line MCF-7 at physiological concentrations and has an antiproliferative effect at supraphysiologic concentrations [43,44,45,46]. DHEAS also has the potential to induce growth of ER-positive/AR-positive cells [47].

It is possible that a greater range of intake would result in further reduction of the hormones, and may lead to a decreased risk of breast cancer. In contrast, flavanones were associated with higher circulating DHEAS level. The higher intake of flavanones (median = 27.41 mg/d) compared to flavan-3-ols (median = 23.16 mg/d) and anthocyanins (median = 10.18 mg/d), may partially offset the lower concentrations shown with higher anthocyanin and flavan-3-ol intake, and more importantly emphasizes that the subclasses should be analyzed separately. Nevertheless, alternative mechanisms are needed to explain the heterogeneity due to menopausal status. Circulating sex hormone levels are relatively high before menopause, and therefore the degree to which dietary flavonoids can act against endogenous sex hormones may be limited; whereas after estrogen levels fall at menopause, the protective effect of flavonoids may become more evident.

Our study has several strengths. In a large sample of premenopausal women we assessed the association between multiple sex hormones and flavonoid intake by subclasses. The sex hormones that fluctuate during the menstrual cycle were timed, that is, measured in both follicular and luteal phases, adding accuracy to the analyses. We were also able to take advantage of high-quality dietary assessment based on validated FFQ, and had detailed data on a range of potentially important covariates. There are also limitations of our study. Measurement errors in the assessment of flavonoid intake could attenuate the associations, if any; meanwhile, the nutrient calculations for food items using the United States Department of Agriculture (USDA) food content database is imperfect. However, the relative accuracy of the exposures in our study is acceptable, since validation of FFQ versus 7-day dietary records suggested that the questionnaire was able to represent the average intake over the yearly period. The measurement error of the sex hormones examined should also be considered. The validity depends on both the assays performed at different labs and the reproducibility of a single measure. Notably, DHEAS, DHEA, and SHBG had the highest ICC among all hormones we assessed. The true associations between flavonoid intake and the other hormones (with lower ICCs) could have been undetectable because they were attenuated by random within-person variation. For samples that were untimed, the Spearman correlations between samples collected during the follicular and luteal phase were 0.60 for DHEA, 0.82 for DHEAS, 0.63 for testosterone, 0.55 for prolactin, and 0.86 for SHBG in this population. Therefore, any fluctuation might also have attenuated the observed association. We did not perform formal multiple testing adjustment, but given the number of statistical tests we conducted, some of the significant associations could be due to chance. If adjusted for multiple comparisons of the hormones, only the association between anthocyanins and DHEA would remain significant. We were also not able to control for all potential confounders, such as other nutrients rich in fruits and vegetables. However, in our study, the Pearson correlation coefficients between total flavonoid intake and intake of dietary fiber, carotenoids, total fruit, and total vegetables range from 0.06 to 0.11, suggesting that confounding by these nutrients/foods seems unlikely. Our subclass analyses, though, suggest that most of the significant association between flavonoids and DHEAS was driven by flavan-3-ol, which is enriched in tea [48,49]. However, in our study, the geometric mean concentrations of the biomarkers of interest did not change significantly by quartiles of tea consumption (Supplementary Materials Table S2), indicating that there is little chance that the observed associations in our analyses was due to other substances in tea. Finally, this is a cross-sectional study by nature, therefore we cannot assess causality.

In conclusion, our study suggests that usual intake of total flavonoids at levels seen in this population had limited impact on circulating sex hormones in premenopausal women. Intake of anthocyanins and flavan-3-ols was associated with lower levels of DHEA and DHEAS, while it is important that the subclasses of flavonoid be considered separately as they may affect sex hormones differently.

## Figures and Tables

**Table 1:** Characteristics at blood draw of premenopausal women in the Nurses’ Health Study II (NHSII, *n* = 1989).

Variables	Total Flavonoid Intake (mg/day)			
	Quartile 1	Quartile 2	Quartile 3	Quartile 4
	<170.3	170.3–241.8	241.9–359.3	>359.3
	*n* = 497	*n* = 496	*n* = 498	*n* = 498
	Mean (SD ^+^)	Mean (SD ^+^)	Mean (SD ^+^)	Mean (SD ^+^)
Age, years	42.3 (4.1)	42.5 (4.2)	43.0 (4.0)	43.0 (3.9)
Caucasian, %	98.0	99.0	98.2	98.0
Height, inches	64. 9 (2.7)	64.8 (2.5)	65.3 (2.5)	64.9 (2.7)
Body mass index (BMI), kg/m^2^	26. 6 (6.7)	26.1 (7.2)	25.2 (6.3)	26.4 (7.5)
BMI at age 18, kg/m^2^	21.2 (3.2)	21.3 (3.2)	20.9 (2.9)	21.1 (3.0)
Physical activity, MET*-hours/week	16.2 (7.2)	17.7 (17.3)	20.0 (18.7)	18.2 (17.2)
Current smoker, %	11.3	7.9	5.8	7.0
AHEI ** score	47.9 (9.5)	52.0 (9.8)	53.9 (10.8)	53.3 (10.7)
Total fruit intake, servings/day	1.2 (0.7)	1.95 (0.9)	2.4 (1.2)	2.2 (1.5)
Total vegetable intake, servings/day	3.1 (1.8)	3.7 (1.9)	4.0 (2.1)	4.0 (2.3)
Age at menarche <12 years, %	23.7	19.6	19.1	25.3
Usual menstrual cycle pattern regular, %	93.1	89.5	92.2	92.7
Parity	2.4 (0.96)	2.3 (0.97)	2.3 (0.93)	2.3 (0.92)
Parous, %	83.7	82.6	76.3	80.5
Age at first birth among parous women, years	26.4 (4.2)	26.7 (4.7)	26.9 (4.4)	26.7 (4.5)
Past breastfeeding history, %	67.8	69.2	64.5	69.5
Past oral contraceptive use, %	86.9	83.7	83.5	86.1
Family history of breast cancer, %	9.3	11.1	9.0	8.2
Benign breast disease history (biospy confirmed), %	16.1	16.9	18.9	16.1

* MET, metabolic equivalent of task, an objective measure of the ratio of the rate at which a person expends energy, relative to the mass of that person while performing some specific physical activity compared to a reference energy expended when sitting quietly (roughly at 3.5 mL of oxygen per kilogram per minute); ** AHEI, the Alternative Healthy Eating Index, ranging from 0 to10 points. A score of 10 indicates that the recommendations were fully met, whereas a score of 0 represents the least healthy dietary behavior. ^+^ Unless otherwise stated. SD, standard deviation.

**Table 2:** Plasma hormone concentrations among premenopausal women in the NHSII (*n* = 1989).

Plasma Hormone	*n*	Median	10th Pctl–90th Pctl
Follicular estradiol, pg/mL	1397	46.8	22.1–101
Luteal estradiol, pg/mL	1524	134	72.5–237
Follicular free estradiol, pg/mL	1361	0.59	0.3–1.18
Luteal free estradiol, pg/mL	1508	1.69	0.93–2.86
Follicular estrone, pg/mL	1417	40.6	25–67.7
Luteal estrone, pg/mL	1571	84.2	51.1–142
Follicular estrone sulfate, pg/mL	444	661	299–1517
Luteal estrone sulfate, pg/mL	449	1454	573–3326
Luteal progesterone, ng/dL	1587	1396	251–2696
DHEA *, ng/dL	476	614	346–1127
DHEAS *, μg/dL	1240	86.8	39.5–163
Testosterone ^+^, ng/dL	1956	23.5	14.2–36.8
Free testosterone ^+^, ng/dL	1898	0.20	0.1–0.37
Androstenedione ^+^, ng/dL	623	100	60.2–164
Prolactin ^+^, ng/dL	1300	14.5	8.24–28.7
SHBG ^+^, nmol/L	1916	64.5	32.4–116

* Include luteal or untimed samples. DHEA: dehydroepiandrosterone; DHEAS: dehydroepiandrosterone sulfate. ^+^ Include average of follicular and luteal samples, or untimed samples. SHBG: sex hormone-binding globulin.

**Table 3:** Adjusted ^1^ geometric mean concentration ^2^ of hormones by categories of average flavonoid consumption among 1989 premenopausal women in the NHSII.

		Total Flavonoid Intake, mg/day						
Hormone	*n*	Quartile 1	Quartile 2	Quartile 3	Quartile 4	*p*-Trend ^3^	Percentage Difference ^4^ and 95% Confidence Interval (CI)	
Range		<170.3	170.3–241.8	241.9–359.3	>359.3		Mean	95% CI
Median Flavonoid intake		128.0	204.9	288.2	550.2			
Follicular estradiol, pg/mL	1397	77.8	79.3	75.5	75.8	0.53	−2.6%	(−13.4%, 9.5%)
Luteal estradiol, pg/mL	1524	129.8	134.9	129.1	129.6	0.65	−0.1%	(−6.9%, 7.2%)
Follicular free estradiol, pg/mL	1361	0.79	0.82	0.75	0.78	0.60	−0.9%	(−10.0%, 9.1%)
Luteal free estradiol, pg/mL	1508	1.58	1.68	1.59	1.59	0.60	0.4%	(−6.8%, 8.1%)
Follicular estrone, pg/mL	1417	47.3	46.5	47.1	46.7	0.84	−1.2%	(−8.1%, 6.2%)
Luteal estrone, pg/mL	1571	81.2	81.6	82.0	79.4	0.38	−2.2%	(−8.3%, 4.3%)
Follicular estrone sulfate, pg/mL	444	777.1	773.2	785.1	795.1	0.77	2.3%	(−16.3%, 25.1%)
Luteal estone sulfate, pg/mL	449	1511.2	1379.4	1761.3	1404.5	0.51	−7.1%	(−24.8%, 14.8%)
Luteal progesterone, ng/dL	1587	1135.5	1112.5	1130.8	1051.1	0.06	−7.4%	(−14.6%, 0.4%)
DHEA, ng/dL	386	841.1	797.7	823.9	855.3	0.55	1.7%	(−11.0%, 16.2%)
DHEAS, μg/dL	1083	107.0	98.4	102.7	95.1	0.02	−11.1%	(−18.6%, −3.0%)
Testosterone, ng/dL	1956	24.3	24.5	24.8	24.4	0.97	0.5%	(−4.2%, 5.4%)
Free testosterone, ng/dL	1898	0.20	0.21	0.20	0.20	0.26	−2.1%	(−8.6%, 4.9%)
Androstenedione, ng/dL	623	130.9	123.8	130.0	129.1	0.92	−1.4%	(−9.6%, 7.6%)
Prolactin, ng/dL	1300	22.0	21.9	21.7	23.1	0.25	5.1%	(−5.5%, 16.8%)
SHBG, nmol/L	1675	75.5	74.8	76.2	78.5	0.11	3.9%	(−1.9%, 10.1%)

^1^ Covariates in the model: race, height, age at blood draw, BMI at blood draw, smoking status, alcohol consumption, parity, age at first birth, age at menarche, usual menstrual cycle length, menstrual cycle pattern, family history of breast cancer, history of biopsy-confirmed benign breast disease, duration of oral contraceptive use, physical activity, luteal day difference (number of days with reference to beginning of next menstruation), total calories, blood draw date, blood draw time of the day, and fasting status at blood draw. ^2^ Geometric mean concentration calculated as the expected hormone or other biomarker levels within each quartile of flavonoid intake, based on the multivariate model where the other covariates are set to the distribution at the population level in the study. ^3^ The cut-off *p*-value for statistical significance is 0.003 if considering multiple testing (α = 0.05). ^4^ Percentage difference: the difference in geometric mean concentration of the hormones between the top versus the bottom quartile presented as percentages of the bottom quartile.

**Table 4:** Adjusted geometric mean concentration of hormones by categories of average consumption of flavonoid subclasses among premenopausal women in the NHSII (*n* = 1989).

**Hormones**	**Flavonol Intake, mg/day**						
	**Quartile1**	**Quartile2**	**Quartile3**	**Quartile4**	***p*-Trend**	**Percentage Difference and 95% CI**	
	2.8–10.9	10.9–14.9	15.0–21.2	>21.2		Mean	95% CI
Median	8.6	12.9	17.5	28.5			
Follicular estradiol, pg/mL	76.9	78.0	79.7	74.1	0.50	−3.7%	(−15.7%, 10.1%)
Luteal estradiol, pg/mL	133.2	130.8	127.0	132.2	0.93	−0.7%	(−8.1%, 7.2%)
Follicular free estradiol, pg/mL	0.77	0.81	0.79	0.77	0.72	0.0%	(−9.3%, 10.3%)
Luteal free estradiol, pg/mL	1.61	1.65	1.57	1.61	0.72	−0.2%	(−7.8%, 8.0%)
Follicular estrone, pg/mL	46.4	47.8	47.2	46.4	0.79	0.1%	(−7.0%, 7.8%)
Luteal estrone, pg/mL	82.1	81.7	80.3	79.9	0.38	−2.8%	(−9.1%, 4.0%)
Follicular estrone sulfate, pg/mL	824.9	753.4	738.9	786.1	0.89	−4.7%	(−23.2%, 18.3%)
Luteal estone sulfate, pg/mL	1497.9	1565.0	1608.9	1445.1	0.55	−3.5%	(−21.1%, 18.0%)
Luteal progesterone, ng/dL	1110.1	1146.1	1101.2	1081.7	0.31	−2.6%	(−10.3%, 5.8%)
DHEA, ng/dL	813.0	853.1	846.7	854.6	0.61	5.1%	(−9.6%, 22.2%)
DHEAS, μg/dL	101.3	103.3	103.0	98.2	0.34	−3.1%	(−11.5%, 6.2%)
Testosterone, ng/dL	24.3	24.8	24.7	24.3	0.80	0.0%	(−4.9%, 5.2%)
Free testosterone, ng/dL	0.20	0.21	0.20	0.20	0.69	−0.4%	(−7.8%, 7.7%)
Androstenedione, ng/dL	128.2	130.2	131.3	129.4	0.89	0.9%	(−8.2%, 10.9%)
Prolactin, ng/dL	22.5	20.9	22.2	23.0	0.33	2.0%	(−8.4%, 13.6%)
SHBG, nmol/L	77.4	72.6	75.9	78.3	0.30	1.2%	(−5.6%, 8.5%)
	**Flavanone Intake, mg/day**						
	**Quartile1**	**Quartile2**	**Quartile3**	**Quartile4**	***p*-Trend**	**Percentage Difference and 95% CI**	
	0.05–14.9	14.9–27.4	27.4–47.4	>47.4		Mean	95% CI
Median	9.0	21.0	35.9	66.6			
Follicular estradiol, pg/mL	75.0	78.2	74.5	79.7	0.52	6.3%	(−7.8%, 22.5%)
Luteal estradiol, pg/mL	135.6	125.5	128.4	134.9	0.60	−0.5%	(−8.3%, 8.0%)
Follicular free estradiol, pg/mL	0.70	0.82	0.83	0.79	0.16	11.8%	(1.5%, 23.2%)
Luteal free estradiol, pg/mL	1.68	1.55	1.60	1.62	0.77	−3.5%	(−11.0%, 4.7%)
Follicular estrone, pg/mL	46.2	46.2	48.2	47.3	0.40	2.4%	(−4.5%, 9.7%)
Luteal estrone, pg/mL	84.7	76.6	80.0	83.3	0.62	−1.7%	(−7.8%, 4.9%)
Follicular estrone sulfate, pg/mL	741.2	757.5	779.6	869.8	0.11	17.4%	(−5.4%, 45.5%)
Luteal estone sulfate, pg/mL	1582.9	1342.3	1479.6	1611.8	0.50	1.8%	(−17.8%, 26.1%)
Luteal progesterone, ng/dL	1089.4	1110.2	1119.5	1122.4	0.52	3.0%	(−5.4%, 12.2%)
DHEA, ng/dL	864.0	841.6	836.3	805.5	0.30	−6.8%	(−18.3%, 6.3%)
DHEAS, μg/dL	98.0	99.2	102.3	106.9	0.04	9.0%	(−0.3%, 19.3%)
Testosterone, ng/dL	24.6	24.4	24.2	24.9	0.63	1.0%	(−4.2%, 6.4%)
Free testosterone, ng/dL	0.20	0.20	0.20	0.20	0.89	0.1%	(−6.3%, 7.0%)
Androstenedione, ng/dL	128.4	128.0	132.3	129.8	0.74	1.2%	(−7.7%, 10.8%)
Prolactin, ng/dL	22.1	21.9	22.8	21.4	0.56	−3.3%	(−12.0%, 6.3%)
SHBG, nmol/L	79.8	77.0	72.7	76.1	0.14	−4.7%	(−10.5%, 1.4%)
	**Flavone Intake, mg/d**						
	**Quartile1**	**Quartile2**	**Quartile3**	**Quartile4**	***p*-Trend**	**Percentage Difference and 95% CI**	
	0.1–1.1	1.1–1.6	1.6–2.3	>2.3		Mean	95% CI
Median	0.8	1.3	1.9	3.0			
Follicular estradiol, pg/mL	81.7	75.2	69.7	82.3	0.82	0.7%	(−13.5%, 17.3%)
Luteal estradiol, pg/mL	133.4	125.5	127.8	136.1	0.35	2.0%	(−6.4%, 11.2%)
Follicular free estradiol, pg/mL	0.74	0.82	0.77	0.81	0.24	9.5%	(−0.7%, 20.9%)
Luteal free estradiol, pg/mL	1.67	1.54	1.59	1.63	0.98	−2.0%	(−10.5%, 7.4%)
Follicular estrone, pg/mL	47.0	46.3	46.0	48.4	0.35	2.9%	(−4.0%, 10.3%)
Luteal estrone, pg/mL	83.4	77.4	78.9	84.2	0.35	0.9%	(−5.7%, 7.9%)
Follicular estrone sulfate, pg/mL	747.9	793.5	794.3	810.1	0.50	8.3%	(−10.5%, 31.1%)
Luteal estone sulfate, pg/mL	1434.7	1563.9	1473.7	1694.5	0.12	18.1%	(−2.7%, 43.4%)
Luteal progesterone, ng/dL	1070.2	1118.1	1114.1	1135.8	0.23	6.1%	(−2.7%, 15.8%)
DHEA, ng/dL	892.3	856.0	813.2	789.4	0.06	−11.5%	(−22.4%, 0.9%)
DHEAS, μg/dL	97.6	100.9	102.3	105.5	0.10	8.1%	(−1.4%, 18.5%)
Testosterone, ng/dL	24.7	24.1	24.4	24.9	0.57	0.8%	(−4.3%, 6.2%)
Free testosterone, ng/dL	0.20	0.20	0.20	0.20	0.92	−0.5%	(−7.1%, 6.5%)
Androstenedione, ng/dL	128.7	128.9	133.6	128.7	0.94	0.0%	(−9.1%, 10.0%)
Prolactin, ng/dL	22.2	22.1	22.5	21.8	0.66	−2.2%	(−11.0%, 7.4%)
SHBG, nmol/L	79.7	76.2	73.1	75.9	0.18	−4.8%	(−11.0%, 1.9%)
	**Flavan-3-ol Intake, mg/day**						
	**Quartile1**	**Quartile2**	**Quartile3**	**Quartile4**	***p*-Trend**	**Percentage Difference and 95% CI**	
	1.84–13.8	13.8–23.2	23.2–47.3	>47.3		Mean	95% CI
Median	10.2	17.5	31.9	92.5			
Follicular estradiol, pg/mL	77.9	74.9	82.3	75.3	0.62	−3.3%	(−14.2%, 9.0%)
Luteal estradiol, pg/mL	133.1	127.3	132.9	127.5	0.38	−4.2%	(−10.9%, 3.0%)
Follicular free estradiol, pg/mL	0.81	0.79	0.75	0.77	0.43	−4.7%	(−14.0%, 5.6%)
Luteal free estradiol, pg/mL	1.62	1.58	1.64	1.58	0.61	−2.3%	(−9.4%, 5.4%)
Follicular estrone, pg/mL	47.3	47.1	46.2	47.0	0.95	−0.7%	(−7.9%, 7.1%)
Luteal estrone, pg/mL	81.1	81.0	83.4	78.8	0.23	−2.9%	(−8.7%, 3.4%)
Follicular estrone sulfate, pg/mL	774.6	762.4	722.1	820.0	0.37	5.9%	(−12.9%, 28.7%)
Luteal estone sulfate, pg/mL	1435.9	1558.6	1641.4	1471.5	0.75	2.5%	(−16.1%, 25.2%)
Luteal progesterone, ng/dL	1130.2	1121.9	1131.1	1044.5	0.03	−7.6%	(−14.8%, 0.2%)
DHEA, ng/dL	814.2	817.5	823.9	897.8	0.11	10.3%	(−4.5%, 27.3%)
DHEAS, μg/dL	107.5	100.8	99.1	95.4	0.01	−11.3%	(−18.3%, −3.7%)
Testosterone, ng/dL	24.2	24.6	24.9	24.1	0.48	−0.5%	(−5.2%, 4.3%)
Free testosterone, ng/dL	0.20	0.21	0.20	0.20	0.49	−0.4%	(−7.2%, 6.8%)
Androstenedione, ng/dL	128.9	126.1	129.9	131.2	0.50	1.8%	(−6.3%, 10.5%)
Prolactin, ng/dL	22.22	21.60	21.36	23.07	0.23	3.8%	(−6.4%, 15.1%)
SHBG, nmol/L	76.2	72.2	77.7	78.1	0.10	2.5%	(−3.4%, 8.8%)
	**Anthocyanin Intake, mg/day**						
	**Quartile1**	**Quartile2**	**Quartile3**	**Quartile4**	***p*-Trend**	**Percentage Difference and 95% CI**	
	0.24–5.4	5.4–10.2	10.2–17.2	>17.2		Mean	95% CI
Median	3.5	7.7	13.4	23.5			
Follicular estradiol, pg/mL	80.7	74.8	75.6	76.5	0.61	−5.3%	(−18.5%, 10.1%)
Luteal estradiol, pg/mL	128.3	135.0	131.3	127.7	0.51	−0.5%	(−8.0%, 7.6%)
Follicular free estradiol, pg/mL	0.80	0.79	0.78	0.74	0.14	−7.3%	(−16.5%, 2.9%)
Luteal free estradiol, pg/mL	1.60	1.66	1.61	1.55	0.22	−3.3%	(−11.1%, 5.2%)
Follicular estrone, pg/mL	46.8	47.8	47.9	44.4	0.09	−5.0%	(−11.7%, 2.1%)
Luteal estrone, pg/mL	76.8	84.3	82.0	80.0	0.78	4.1%	(−2.3%, 10.9%)
Follicular estrone sulfate, pg/mL	816.0	843.8	789.2	678.3	0.02	−16.9%	(−32.5%, 2.4%)
Luteal estone sulfate, pg/mL	1375.6	1733.9	1491.5	1454.5	0.69	5.7%	(−11.8%, 26.7%)
Luteal progesterone, ng/dL	1074.6	1156.7	1123.6	1080.7	0.63	0.6%	(−7.7%, 9.5%)
DHEA, ng/dL	922.7	841.1	822.3	757.0	0.003	−18.0%	(−27.9%, −6.7%)
DHEAS, μg/dL	99.4	104.3	100.1	100.4	0.85	1.0%	(−8.3%, 11.3%)
Testosterone, ng/dL	24.3	24.4	24.4	25.1	0.19	3.3%	(−1.8%, 8.6%)
Free testosterone, ng/dL	0.20	0.20	0.21	0.20	0.41	−2.8%	(−9.6%, 4.5%)
Androstenedione, ng/dL	132.4	130.8	131.8	125.0	0.18	−5.6%	(−13.6%, 3.2%)
Prolactin, ng/dL	22.0	23.6	21.7	21.6	0.31	−1.9%	(−11.4%, 8.6%)
SHBG, nmol/L	76.5	74.9	76.4	77.4	0.48	1.2%	(−5.1%, 7.9%)
	**Isoflavone Intake, mg/day**						
	**Quartile1**	**Quartile2**	**Quartile3**	**Quartile4**	***p*-Trend**	**Percentage Difference and 95% CI**	
	0.04–0.27	0.27–0.43	0.43–0.88	>0.88		Mean	95% CI
Median	0.2	0.3	0.5	2.2			
Follicular estradiol, pg/mL	75.3	77.6	80.4	76.4	0.84	1.5%	(−12.5%, 17.9%)
Luteal estradiol, pg/mL	129.1	135.4	131.6	126.9	0.20	−1.7%	(−8.6%, 5.7%)
Follicular free estradiol, pg/mL	0.78	0.77	0.81	0.78	0.93	1.1%	(−8.4%, 11.5%)
Luteal free estradiol, pg/mL	1.62	1.65	1.61	1.54	0.10	−4.8%	(−12.5%, 3.7%)
Follicular estrone, pg/mL	47.6	45.1	48.4	46.3	0.61	−2.9%	(−9.2%, 3.9%)
Luteal estrone, pg/mL	80.8	81.9	81.7	79.7	0.41	−1.4%	(−7.4%, 5.0%)
Follicular estrone sulfate, pg/mL	700.9	833.7	817.9	881.1	0.12	25.7%	(3.6%, 52.6%)
Luteal estone sulfate, pg/mL	1481.1	1478.6	1554.6	1600.4	0.44	8.1%	(−13.0%, 34.2%)
Luteal progesterone, ng/dL	1067.1	1105.0	1156.1	1134.8	0.39	6.3%	(−2.2%, 15.7%)
DHEA, ng/dL	867.7	773.8	904.5	775.2	0.09	−10.7%	(−21.4%, 1.5%)
DHEAS, μg/dL	93.5	102.7	109.0	105.4	0.14	12.7%	(3.5%, 22.8%)
Testosterone, ng/dL	24.7	23.8	25.2	24.1	0.42	−2.4%	(−7.2%, 2.7%)
Free testosterone, ng/dL	0.21	0.19	0.21	0.20	0.35	−4.2%	(−11.5%, 3.7%)
Androstenedione, ng/dL	131.0	122.4	132.4	130.0	0.79	−0.8%	(−9.3%, 8.5%)
Prolactin, ng/dL	22.9	21.5	21.7	22.2	0.96	−3.0%	(−12.4%, 7.3%)
SHBG, nmol/L	75.9	77.2	74.9	76.9	0.66	1.3%	(−4.8%, 7.8%)

## Supplementary Materials

The following are available online at https://www.mdpi.com/2072-6643/11/11/2669/s1: Table S1: Pearson correlation coefficients of flavonoid subclasses. Table S2: Adjusted geometric mean concentration of hormones by categories of tea consumption in 1989 premeopausal women in NHSII. Table S3: Main food sources of total flavonoid and flavonoid subclasses in NHSII.
