# Supplementary material for: Flavonoid Intake and Plasma Sex Steroid Hormones, Prolactin, and Sex Hormone-Binding Globulin in Premenopausal Women
Source: Nutrients. 2019 Nov 5;11(11):2669. doi: 10.3390/nu11112669 (PMC6928816; doi:10.3390/nu11112669)
Supplement: Supplementary file 1 [file nutrients-11-02669-s001.docx]

**Table S1.**  Pearson correlation coefficients of flavonoid subclasses.

| **Variables** | **Total flavonoid** | **Flavonol** | **Flavone** | **Flavanone** | **Flavan-3-ol** | **Anthocyanidin** | **Isoflavone** |
| --- | --- | --- | --- | --- | --- | --- | --- |
| Total flavonoid | 1 | 0.7833 | 0.14945 | 0.13762 | 0.97342 | 0.18146 | 0.02485 |
|  |  | <.0001 | <.0001 | <.0001 | <.0001 | <.0001 | 0.268 |
|  |  |  |  |  |  |  |  |
| Flavonol |  | 1 | 0.18038 | 0.05962 | 0.74867 | 0.18701 | 0.0816 |
|  |  |  | <.0001 | 0.0078 | <.0001 | <.0001 | 0.0003 |
|  |  |  |  |  |  |  |  |
| Flavone |  |  | 1 | 0.87518 | -0.00957 | 0.22842 | 0.02401 |
|  |  |  |  | <.0001 | 0.6699 | <.0001 | 0.2845 |
|  |  |  |  |  |  |  |  |
| Flavanone |  |  |  | 1 | -0.01046 | 0.11924 | -0.01137 |
|  |  |  |  |  | 0.641 | <.0001 | 0.6124 |
|  |  |  |  |  |  |  |  |
| Flavan-3-ol |  |  |  |  | 1 | 0.06343 | 0.00905 |
|  |  |  |  |  |  | 0.0047 | 0.6868 |
|  |  |  |  |  |  |  |  |
| Anthocyanidin |  |  |  |  |  | 1 | 0.09559 |
|  |  |  |  |  |  |  | <.0001 |
|  |  |  |  |  |  |  |  |
| Isoflavone |  |  |  |  |  |  | 1 |

**Table S2.** Adjusted geometric mean concentration of hormones by categories of tea consumption in 1989 premeopausal women in NHSII.

|  | Tea consumption, servings/d | | | |  |  |  |
| --- | --- | --- | --- | --- | --- | --- | --- |
|  | Quartile1 | Quartile2 | Quartile3 | Quartile4 | P trend | Percentage difference and 95% CI | |
|  | < 2 servings/month | 2 servings/month ~3 servings/week | 3 servings/week ~1 serving/day | > 1 serving/day |  | Mean | 95% CI |
| Median intake, servings/d | 0 | 0.1 | 0.6 | 1.8 |  |  |  |
| Follicular estradiol, pg/mL | 78.9 | 76.8 | 75.4 | 77.4 | 0.91 | -1.9% | (-15.1%, 13.2%) |
| Luteal estradiol, pg/mL | 130.9 | 130.1 | 131.0 | 131.2 | 0.87 | 0.2% | (-6.9%, 7.8%) |
| Follicular free estradiol, pg/mL | 0.8 | 0.8 | 0.8 | 0.8 | 0.87 | -2.3% | (-12.1%, 8.5%) |
| Luteal free estradiol, pg/mL | 1.6 | 1.6 | 1.7 | 1.6 | 0.61 | -3.0% | (-10.0%, 4.4%) |
| Follicular estrone, pg/mL | 48.3 | 46.7 | 46.8 | 46.0 | 0.29 | -4.8% | (-11.7%, 2.6%) |
| Luteal estrone, pg/mL | 83.8 | 80.5 | 81.2 | 79.2 | 0.19 | -5.5% | (-11.6%, 1.0%) |
| Follicular estrone sulfate, pg/mL | 846.3 | 745.3 | 786.2 | 785.5 | 0.86 | -7.2% | (-24.3%, 13.8%) |
| Luteal estone sulfate, pg/mL | 1503.7 | 1500.7 | 1606.0 | 1443.3 | 0.61 | -4.0% | (-20.3%, 15.7%) |
| Luteal progesterone, ng/dL | 1079.1 | 1133.0 | 1074.7 | 1129.9 | 0.55 | 4.7% | (-3.8%, 13.9%) |
| DHEA, ng/dL | 847.8 | 790.7 | 921.4 | 815.0 | 0.84 | -3.9% | (-16.7%, 10.9%) |
| DHEAS, ng/dL | 105.5 | 102.5 | 101.7 | 97.0 | 0.07 | -8.0% | (-15.9%, 0.5%) |
| Testosterone, ng/dL | 24.3 | 25.2 | 24.3 | 24.3 | 0.42 | 0.1% | (-4.9%, 5.3%) |
| Free testosterone, ng/dL | 0.2 | 0.2 | 0.2 | 0.2 | 0.10 | -4.1% | (-11.0%, 3.4%) |
| Androstenedione, ng/dL | 125.8 | 132.4 | 135.0 | 125.1 | 0.41 | -0.6% | (-9.2%, 8.9%) |
| Prolactin, ng/dL | 22.9 | 21.2 | 22.0 | 22.2 | 0.84 | -3.0% | (-13.0%, 8.2%) |
| SHBG, nmol/L | 75.1 | 75.9 | 74.1 | 79.5 | 0.06 | 5.8% | (-0.7%, 12.7%) |

**Table S3.** Main food sources of total flavonoid and flavonoid subclasses in NHSII.

|  | Top food items making up the nutrient and its percentage contribution | | | | | |
| --- | --- | --- | --- | --- | --- | --- |
| Total flavonoid | Tea | 34.93% | Apple | 13.41% | Orange juice | 7.53% |
| Flavonol | Onion | 20.61% | Tea | 18.86% | Apple | 10.17% |
| Flavone | Orange juice | 33.49% | Orange | 13.56% | Orange juice w/ calcium | 10.80% |
| Flavanone | Ornage juice | 43.84% | Orange | 23.16% | Orange juice w/ calcium | 14.14% |
| Flavan-3-ol | Tea | 63.56% | Apple | 7.44% | Banana | 7.07% |
| Anthocyanin | Blueberry | 39.87% | Strawberry | 18.36% | Raisin | 12.61% |
| Isoflavone | Tofu | 23.05% | Other | 20.19% | Soymilk | 15.84% |
